# Supplementary material for: Mice with endogenous TDP‐43 mutations exhibit gain of splicing function and characteristics of amyotrophic lateral sclerosis
Source: EMBO J. 2018 May 15;37(11):e98684. doi: 10.15252/embj.201798684 (PMC5983119; doi:10.15252/embj.201798684)
Supplement: Supplementary file 1 — Appendix [file EMBJ-37-e98684-s001.pdf]

Table of Contents

Appendix Figure S1- Turbidity assay shows changes in *LCDmut* **2**

Appendix Figure S2 – CFTR minigene splicing assay is responsive to TDP-43 knockdown. **3**

Appendix Figure S3 – TDP-43 protein levels are unchanged in *RRM2mut* (Left) and *LCDmut* (Right) MEFs. **3**

Appendix Figure S4 – No changes in *LCDmut* RNA binding **4**

Appendix Figure S5 – TDP-43 knock down in MEFs. **5**

Appendix Figure S6 – MA plot for *RRM2mut* embryonic and *LCDmut* adult spinal cord datasets. **6**

Appendix Figure S7 – Splicing analysis comparison sample permutations and true sample ordering. **7**

Appendix Figure S8 – *LCDmut* show no TDP-43 mislocalisation. **8**

Appendix Figure S9 – TDP-43 autoregulation in *RRM2mut* and *LCDmut.* **9**

Appendix Figure S10 – *PACRGL* and *ANKRD42* skiptic exons appear to be unchanged in mutant vs controls. **10**

Appendix Table S1 **11**

Appendix Table S2 **12**

Appendix Table S3 **14**

Appendix Table S4 **15**

Appendix Table S5 **17**

Appendix Table S6 **17**

Supplementary materials and methods **18**

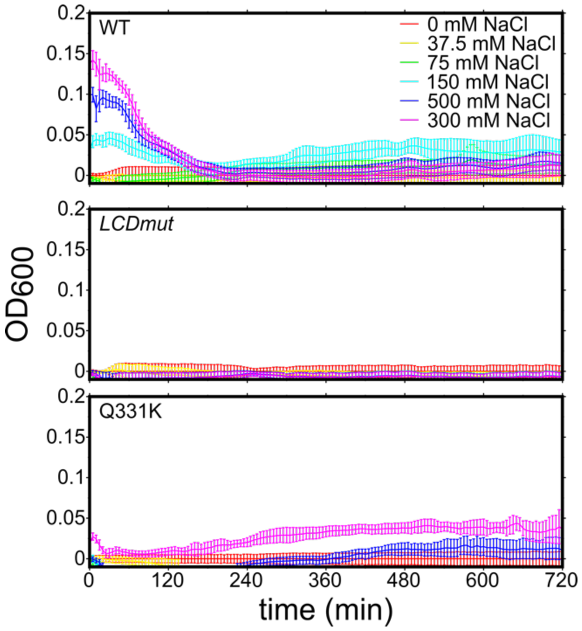

Appendix Figure S1- Turbidity assay shows changes in *LCDmut*

Phase separation as measured by turbidity for 20 µM WT, *LCDmut*, and Q331K TDP-43 C-terminal fragments from residue 267 to 414 in the presence of 0-500 mM NaCl quantified by optical density at 600 nm wavelength light. Measurements were taken in 5 minute intervals over a 12 hour time period. Error bars represent SD of three replicates.

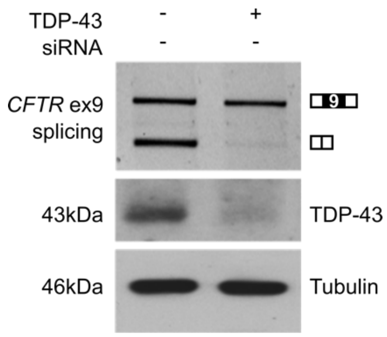

Appendix Figure S2 – CFTR minigene splicing assay is responsive to TDP-43 knockdown.
Agarose gel of CFTR minigene splicing assay shows TDP-43 silencing induces an increase of exon 9 inclusion (top panel). Western blots for TDP-43 (middle panel) and Tubulin (bottom panel) confirm TDP-43 knock-down, whilst Tubulin is unchanged.

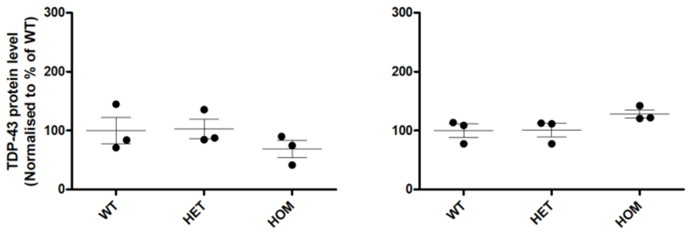

Appendix Figure S3 – TDP-43 protein levels are unchanged in *RRM2mut* (Left) and *LCDmut* (Right) MEFs.
TDP-43 protein ratios relative to GAPDH or tubulin are normalised to the mean of WT (100%). ANOVA p=0.4 (RRM2mut); p=0.16 (LCDmut). Wildtype (WT); heterozygous (HET); homozygous (HOM). Mean and SD are plotted.

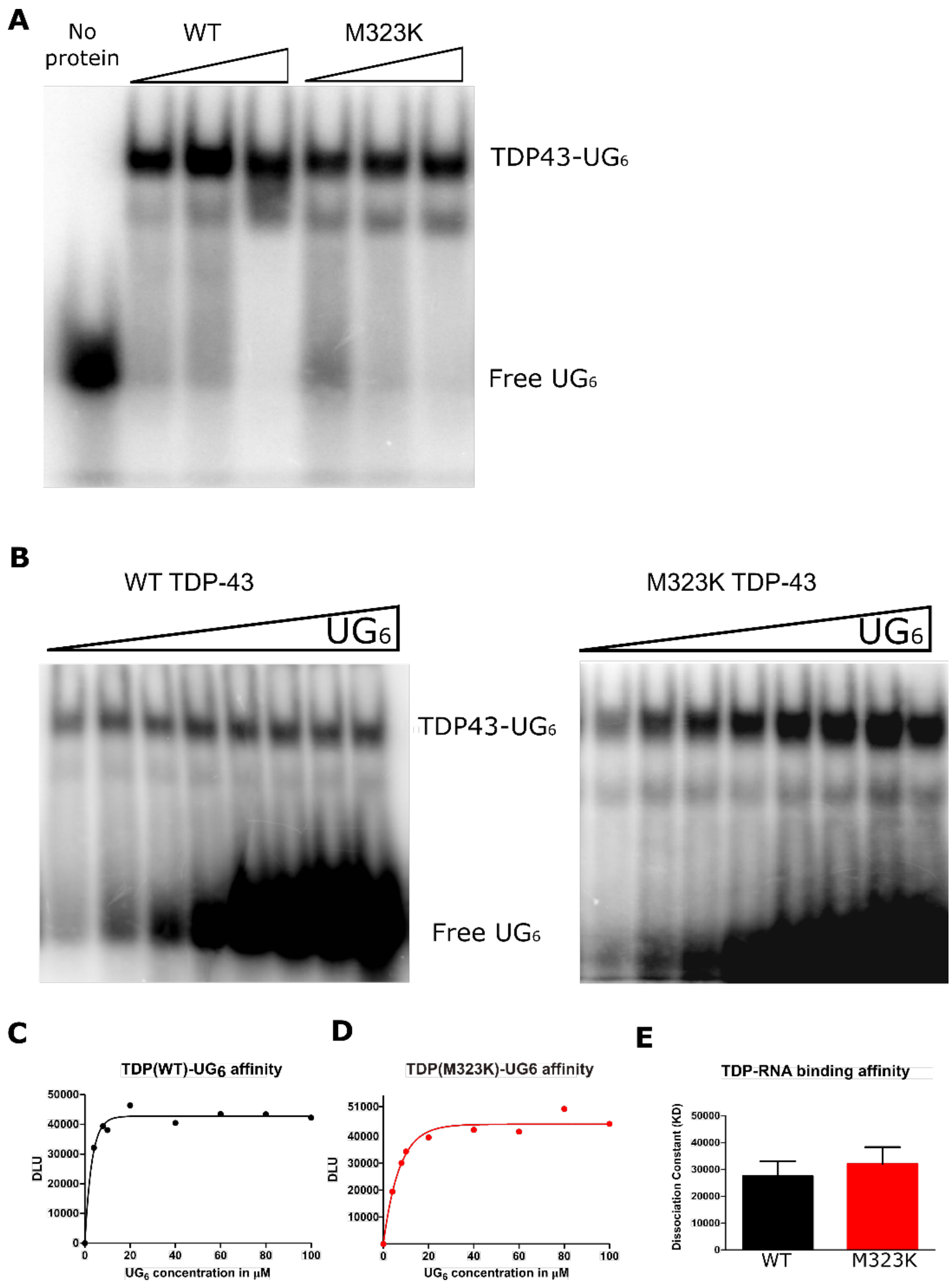

Appendix Figure S4 – No changes in *LCDmut* RNA binding

(**A**) Qualitative EMSA analysis of increasing levels of recombinant TDP-43 protein (250ng, 500ng, 1ug WT and M323K (*LCDmut*) against a fixed amount (0.5ng) of labelled UG_6 RNA repeats show no difference in binding. (**B**) Semi-quantitative EMSA analysis of a fixed amount of recombinant TDP-43 protein (50ng WT and *LCDmut*) against increasing amount of UG_6 RNA repeats (2ng, 4ng, 8ng, 10ng, 20ng, 40ng, 60ng, 80ng). Quantification of WT (**C**) and *LCDmut* (**D**) binding from **B**. DLU: Digital light units. (**E**) Dissociation constant (KD) calculated from B show no differences between WT and *LCDmut*. Mean KD TDP-WT=27602.9±5487.3 (n=3) and TDP-M323K=32163.3±6191.9 (n=2) p=0.62, ANOVA.

A

Lentivirus	shRNA
Tardbp	*TGGATGAGACAGATGCTTC*
Scramble	*CTCGCTTGGGCGAGAGTAA*

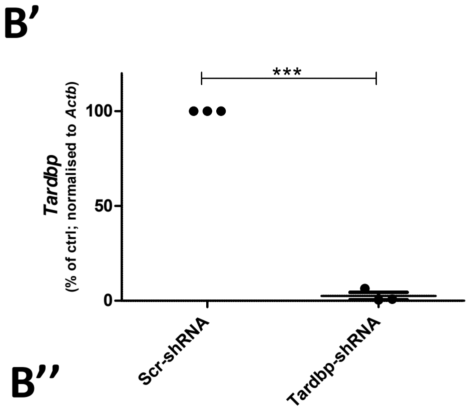

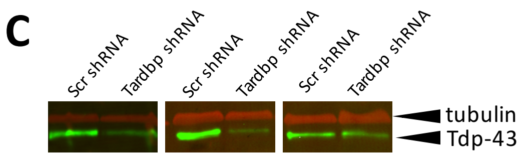

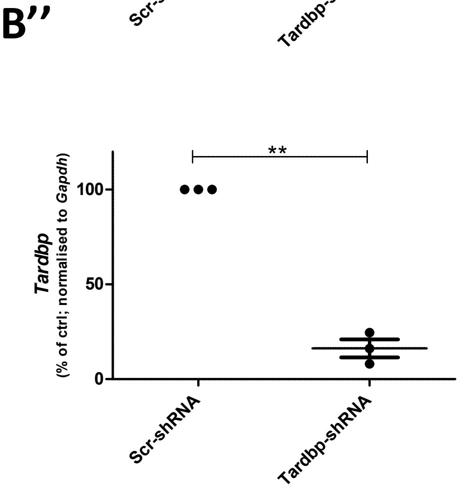

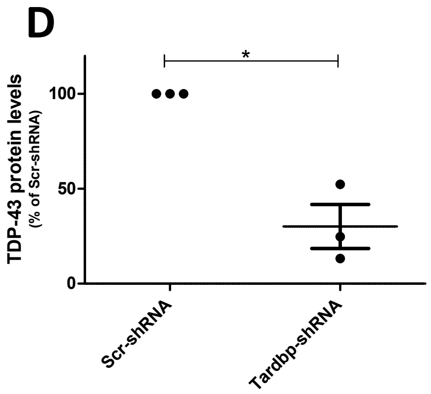

Appendix Figure S5 – TDP-43 knock down in MEFs.
(**A**) Sequence of *Scramble* and *Tardbp* shRNA, that shares homology between mouse and human, obtained from the from the GIPZ lentiviral library. (**B'** and **B''**) *Tardbp* mRNA levels normalised to *Gapdh* (**B'**) and *Actb* (**B''**) are significantly decreased after shRNA treatment. Three experiments are plotted, and *Tardbp* shRNA is normalised to its scr-shRNA control. Two-tailed t-test: B' p=0.0004; B'' p=0.0032 (**C**) Western blot and (**D**) quantification from same experiments show significant reduction of TDP-43 protein normalised to β-tubulin. Two tailed paired t-test, p=0.265. *<0.05; **<0.01; ***<0.001. Mean and SEM are plotted, all experiments N=3.

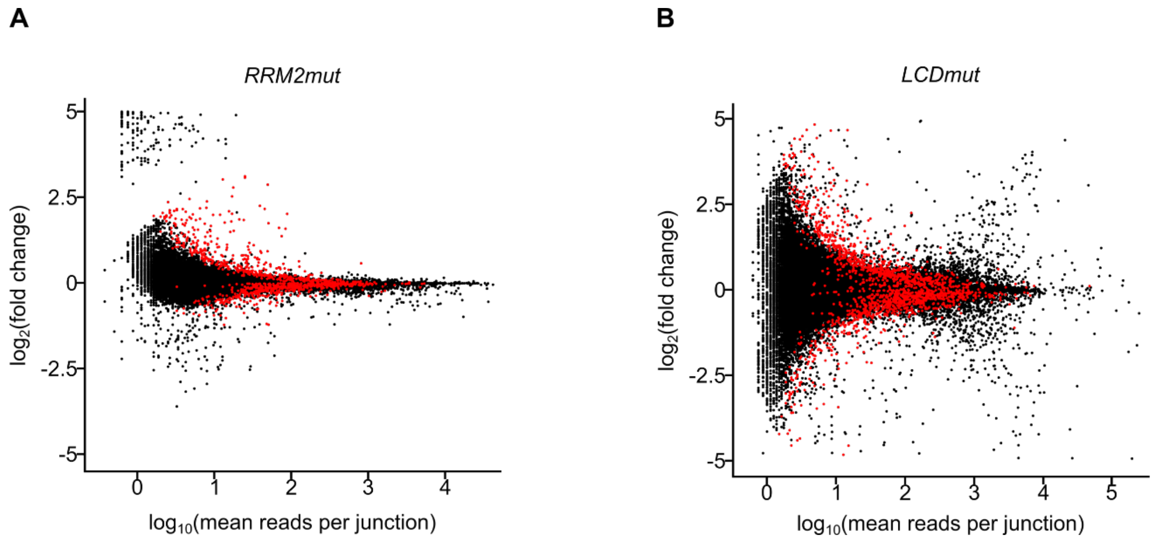

Appendix Figure S6 – MA plot for *RRM2mut* embryonic and *LCDmut* adult spinal cord datasets.
Each splicing event is represented as a binary outcome, of either exclusion or inclusion. For each outcome the mean number of reads that support each outcome (log10) are plotted against the fitted fold change between homozygous mutants and littermate controls. Events that are significant at FDR < 0.05 are highlighted in red.

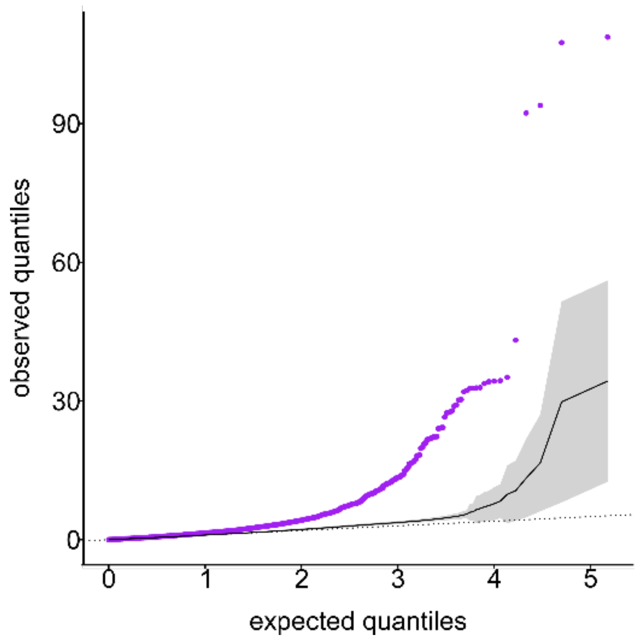

Appendix Figure S7 – Splicing analysis comparison sample permutations and true sample ordering. We permuted the sample order of the 4 wildtype and 4 *LCDmut* homozygotes 100 times to get all possible permutations and re-ran the splicing analysis. Quantile-Quantile plots show the relationship between the expected distribution of -log10(P-values) with those observed in an analysis. The true ordering of wildtype vs *LCDmut* is shown in purple and those of the permuted orderings are shown in grey (mean +- standard deviation). The strong deviation of the true ordering from the permutations demonstrates that the two groups of mice are strongly different and this is what produces the large number of splicing changes, rather than random chance due to the relatively small sample size.

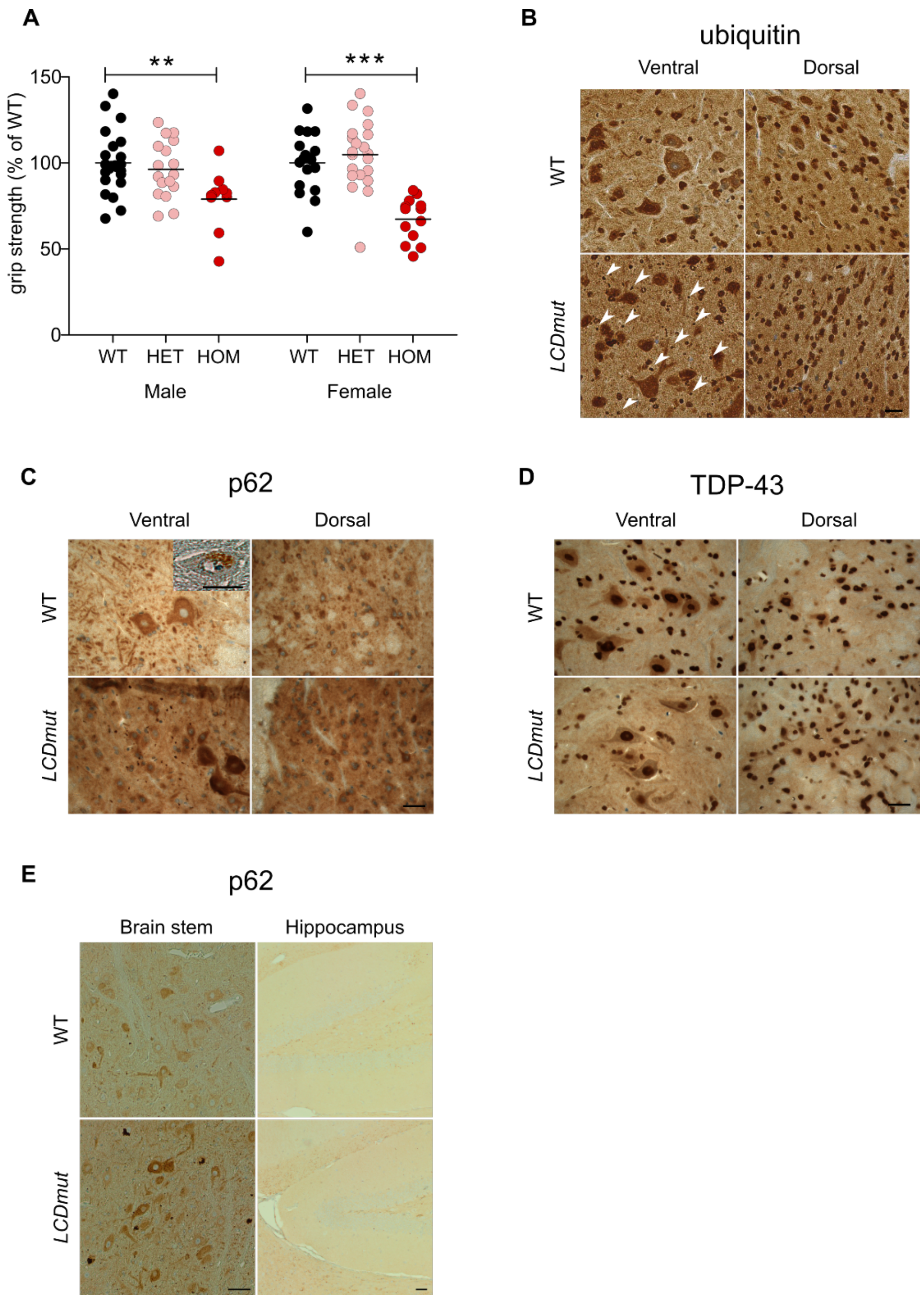

Appendix Figure S8 – *LCDmut* show no TDP-43 mislocalisation.
(**A**) *LCDmut* homozygous male and female mice show significant grip strength deficit (21% and 32% respectively) at 1 year whilst no change is present in heterozygous animals. P=0.0080, males; p<0.0001, females; ANOVA and Bonferroni's Multiple Comparison Test. (**B**) Ubiquitin and (**C**) p62 staining shows multiple ventral horn inclusions in *LCDmut* homozygous (HOM) compared to WT; p62 positive control inserted in box. (**D**) TDP-43 stainings show no cytoplasmic inclusions or nuclear depletion of TDP-43 are present in ventral and dorsal spinal cord from 1 year old *LCDmut* mice and controls. (**E**) p62 staining in brain shows multiple inclusions in *LCDmut* brain stem motor nuclei (left), whilst no change in hippocampus (right).

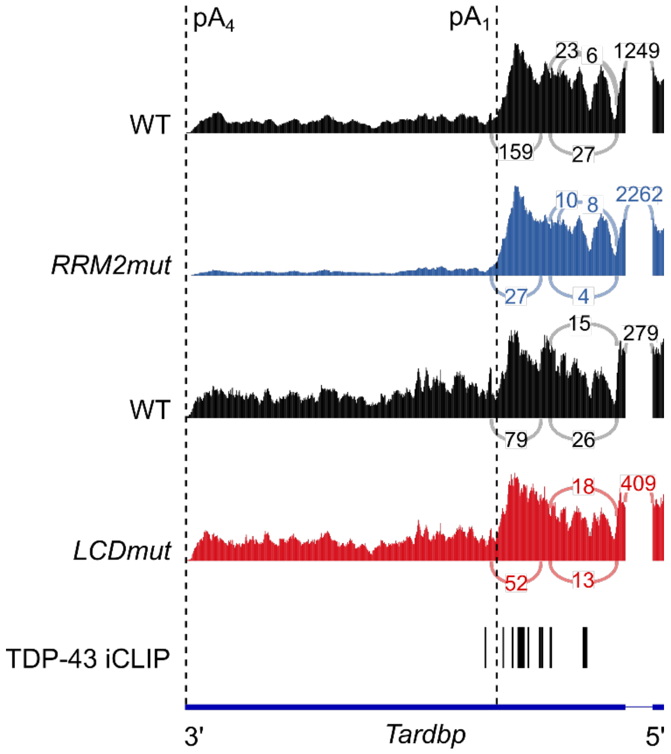

Appendix Figure S9 – TDP-43 autoregulation in *RRM2mut* and *LCDmut.*
Sashimi plots of the *Tardbp* 3'UTR in *RRM2mut, LCDmut* and respective WT control RNA-seq datasets, alongside with Tdp-43 iCLIP trace, illustrate the relation between Tdp-43 binding and the 3'UTR processing events.

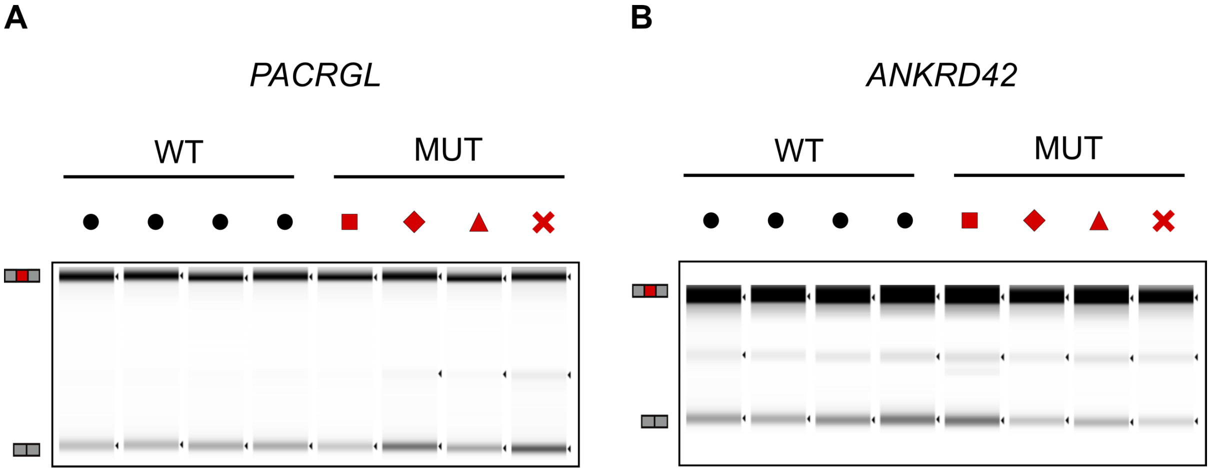

Appendix Figure S10 – *PACRGL* and *ANKRD42* skiptic exons appear to be unchanged in mutant vs controls.

TapeStation images of three primer RT-PCRs amplifying SEs and exon inclusion for *PACRGL* (A) and *ANKRD42* (B). *HERC2*,*UBE3C* and *PEX16* did not show SEs in either *TARDBP* mutant fibroblasts or controls.

Appendix Table S1 – Survival at weaning of *LCDmut* and *RRM2mut* mice in C57BL/6J pure background and C57BL/6J - DBA mixed background. All data from intercrosses between heterozygous parents for each mutation.

LCDmut on C57BL/6J background:

	**Weaning**
**WT**	**23**
**Het**	**25**
**HOM**	**0**

LCDmut on C57BL/6J - DBA background:

	**Weaning**
**WT**	**39**
**Het**	**66**
**HOM**	**30**

RRM2mut on C57BL/6J background:

	**Weaning**
**WT**	**20**
**Het**	**23**
**HOM**	**0**

RRM2mut on C57BL/6J - DBA background:

	**Weaning**
**WT**	**15**
**Het**	**29**
**HOM**	**0**

Appendix Table S2 – List of exons showing significance in the 2-way comparisons of Figure 2.

TDP-43 KD vs RRM2mut						
**Gene name**	**EnsemblID**	**Chromosome**	**Start**	**End**	**TDP-KD z-score**	***RRM2* z-score**
Dnajc5	ENSMUSG00000000826	chr2	181548926	181549000	5.7	6.6
Smg5	ENSMUSG00000001415	chr3	88340720	88340858	5.5	4.6
Zwint	ENSMUSG00000019923	chr10	72673966	72674324	-5.0	-4.4
Sptbn1	ENSMUSG00000020315	chr11	30106785	30109088	-7.2	-6.5
Vamp2	ENSMUSG00000020894	chr11	69090691	69092384	-8.1	-6.5
Ubtf	ENSMUSG00000020923	chr11	102310926	102311036	4.5	5.7
Tmem2	ENSMUSG00000024754	chr19	21780171	21780252	44.8	4.6
Tfpi	ENSMUSG00000027082	chr2	84473943	84474174	5.2	6.7
Mthfsd	ENSMUSG00000031816	chr8	121097937	121098707	6.3	6.2
Arhgap42	ENSMUSG00000050730	chr9	9147356	9148254	5.2	4.8
Map6	ENSMUSG00000055407	chr7	99317669	99320352	4.7	5.4
Palm2+Pakap+Akap2	ENSMUSG00000089945+ENSMUSG00000090053+ENSMUSG00000038729	chr4	57894719	57896982	-4.3	-5.3
TDP-43 KD vs LCDmut						
Gene name	**EnsemblID**	**Chromosome**	**Start**	**End**	**TDP-KD z-score**	***LCDmut* z-score**
Psmd11	ENSMUSG00000017428	chr11	80471642	80471709	-4.9	3.5
Ppp2r5c	ENSMUSG00000017843	chr12	110580401	110583061	-5.2	4.4
Utrn	ENSMUSG00000019820	chr10	12463286	12463360	-4.2	4.2
Hspa4	ENSMUSG00000020361	chr11	53259814	53261815	6.6	-4.7
Speg	ENSMUSG00000026207	chr1	75431259	75432304	4.5	-4.9
Col5a1	ENSMUSG00000026837	chr2	28037016	28039514	4.3	-3.4
Mthfsd	ENSMUSG00000031816	chr8	121097937	121098707	6.3	-4.0
Map6	ENSMUSG00000055407	chr7	99317669	99320352	4.7	-6.2
RRM2mut vs LCDmut						
Gene name	**Ensembl ID**	**Chromosome**	**Start**	**End**	***RRM2* z-score**	***LCDmut* z-score**
H19+Mir675	ENSMUSG00000000031+ENSMUSG00000076275	chr7	142577413	142577640	4.0	-4.4
Uba1	ENSMUSG00000001924	chrX	20662898	20663125	4.1	-4.7
Sri	ENSMUSG00000003161	chr5	8056562	8056607	5.0	-3.5
Sri	ENSMUSG00000003161	chr5	8056608	8056639	5.2	-4.7
Bax	ENSMUSG00000003873	chr7	45466742	45466898	4.7	-3.8
Eif4g2+Gm23262	ENSMUSG00000005610+ENSMUSG00000088948	chr7	111080917	111080964	4.4	-4.8

Eif4g2+Gm23262	ENSMUSG00000005610+ENSMUSG00000088948	chr7	111081507	111081571	4.0	-5.2
Atp6v1b2	ENSMUSG00000006273	chr8	69088768	69088786	5.0	-3.9
Atp6v1b2	ENSMUSG00000006273	chr8	69088787	69088788	5.2	-4.6
Elk3	ENSMUSG00000008398	chr10	93310970	93311135	5.4	-4.4
Rbm25	ENSMUSG00000010608	chr12	83664000	83664259	4.4	-3.4
Tbrg1	ENSMUSG00000011114	chr9	37656998	37657158	4.4	-5.1
Ehmt2	ENSMUSG00000013787	chr17	34905203	34905239	4.5	-4.0
Hnrnpl	ENSMUSG00000015165	chr7	28810940	28811168	5.1	-4.5
Sparc	ENSMUSG00000018593	chr11	55419899	55419900	4.3	-4.6
Sparc	ENSMUSG00000018593	chr11	55419901	55420080	5.1	-4.8
Atp5g3	ENSMUSG00000018770	chr2	73908447	73908707	-4.8	4.3
Sptbn1	ENSMUSG00000020315	chr11	30267948	30268169	4.3	-4.3
Rtn4	ENSMUSG00000020458	chr11	29740998	29741050	-4.0	3.6
Strn3	ENSMUSG00000020954	chr12	51691422	51691914	4.6	-5.8
Papola	ENSMUSG00000021111	chr12	105784746	105784756	4.0	-3.8
Fstl1	ENSMUSG00000022816	chr16	37793888	37794238	-4.9	3.8
Fads2	ENSMUSG00000024665	chr19	10101223	10101503	4.4	-4.5
Mgea5	ENSMUSG00000025220	chr19	45782932	45783520	4.1	-3.5
Caprin1	ENSMUSG00000027184	chr2	103797463	103797599	5.2	-3.8
Ybx1	ENSMUSG00000028639	chr4	119294204	119294480	4.7	-3.6
Rragc	ENSMUSG00000028646	chr4	123935547	123936993	4.4	-3.3
Rpl22	ENSMUSG00000028936	chr4	152332481	152334070	4.0	-4.0
Eif3f	ENSMUSG00000031029	chr7	108934417	108934807	4.7	-4.2
Plp2	ENSMUSG00000031146	chrX	7671314	7671372	4.8	-6.4
Morf4l2	ENSMUSG00000031422	chrX	136741210	136741276	-4.3	5.3
Mthfsd	ENSMUSG00000031816	chr8	121097937	121098707	6.2	-4.0
Igfbp7	ENSMUSG00000036256	chr5	77407540	77408040	5.1	-3.7
Chmp4b	ENSMUSG00000038467	chr2	154657018	154657371	4.3	-3.5
Gm28062+Hnrnpu+Gm16586	ENSMUSG00000039630+ENSMUSG00000100725+ENSMUSG00000089788	chr1	178331866	178331954	-4.1	4.8
Gm28062+Hnrnpu+Gm16586	ENSMUSG00000039630+ENSMUSG00000100725+ENSMUSG00000089788	chr1	178332103	178332356	-5.7	7.3
Ppp1ca	ENSMUSG00000040385	chr19	4192158	4192271	4.8	-5.1
Ckap4	ENSMUSG00000046841	chr10	84533447	84533871	4.4	-4.5
Bri3	ENSMUSG00000047843	chr5	144255234	144255519	4.5	-4.4
Cyp20a1	ENSMUSG00000049439	chr1	60352121	60352170	4.3	-4.8
Map6	ENSMUSG00000055407	chr7	99317669	99320352	5.4	-6.2
Clasp1	ENSMUSG00000064302	chr1	118609501	118612678	5.0	-5.6
Akt1s1+Mir707	ENSMUSG00000076051+ENSMUSG00000011096	chr7	44849344	44849348	4.6	-3.3

Appendix Table S3 – Skiptic exons identified in *LCDmut*.

Chr	*Start*	*End*	*Gene Name*	*WT average PSI*	*LCDmut average PSI*	*ΔPSI*	*PSI Fold Change*	*P-value*
chr2	1.45E+08	1.45E+08	*Dzank1*	0.9795	0.8160	-0.1635	8.9942	1.57E-45
chr7	56157784	56163742	*Herc2*	0.9983	0.7828	-0.2156	128.6068	4.71E-42
chr5	29597184	29600641	*Ube3c*	0.9894	0.7449	-0.2445	24.1694	1.62E-40
chr10	78284350	78287847	*Agpat3*	0.9964	0.9011	-0.0952	27.1469	1.73E-32
chr5	36947237	36952351	*Ppp2r2c*	0.9993	0.9359	-0.0634	94.4229	1.71E-26
chr1	1.18E+08	1.18E+08	*Tsn*	0.9978	0.9012	-0.0965	44.3227	7.36E-23
chr6	1.13E+08	1.13E+08	*Creld1*	1.0000	0.8747	-0.1253	>150	1.05E-22
chr12	50365712	50383400	*Prkd1*	0.9804	0.4517	-0.5288	28.0231	4.99E-18
chr4	19618357	19621928	*Wwp1*	0.9984	0.9111	-0.0873	56.7242	2.13E-17
chr4	58817550	58820128	*AI314180*	0.9981	0.9037	-0.0944	51.0528	1.49E-16
chr19	38265097	38283970	*Lgi1*	0.9847	0.8165	-0.1682	11.9947	1.09E-14
chr11	1.01E+08	1.01E+08	*Psme3*	0.9632	0.8737	-0.0895	3.4328	2.61E-14
chr7	56184707	56185835	*Herc2*	0.9881	0.8836	-0.1045	9.8083	1.25E-13
chr5	1.09E+08	1.09E+08	*Tmem175*	0.9514	0.7273	-0.2241	5.6106	6.73E-12
chr11	45852192	45884332	*Clint1*	0.9962	0.8842	-0.1120	30.5766	1.85E-10
chr10	7710647	7712501	*Lats1*	0.9959	0.9221	-0.0738	19.0151	5.18E-09
chr11	97242047	97244428	*Npepps*	0.9954	0.9425	-0.0530	12.6145	5.99E-09
chr11	1E+08	1E+08	*Nt5c3b*	0.9709	0.8464	-0.1246	5.2820	1.21E-07
chr17	22428565	22446797	*Zfp946*	0.9530	0.6858	-0.2672	6.6817	5.79E-07
chr2	1.31E+08	1.31E+08	*4930402H24Rik*	0.9908	0.9386	-0.0521	6.6376	7.23E-07
chr13	13638376	13641067	*Lyst*	0.9909	0.8287	-0.1622	18.8410	8.97E-07
chr6	30439865	30444427	*Klhdc10*	0.9721	0.8957	-0.0764	3.7346	1.97E-06
chr19	40340146	40344356	*Sorbs1*	0.9877	0.8305	-0.1572	13.7569	2.29E-06
chr5	8143132	8145553	*Adam22*	0.9881	0.8977	-0.0904	8.5959	4.96E-06
chr14	18630431	18888266	*Ube2e2*	0.9542	0.8915	-0.0627	2.3700	8.43E-06
chr7	1.26E+08	1.26E+08	*Xpo6*	0.9657	0.8754	-0.0903	3.6328	2.37E-05
chr8	1.05E+08	1.05E+08	*Cbfb*	0.9736	0.8752	-0.0984	4.7209	4.06E-05
chr6	8216470	8224555	*Mios*	0.9674	0.9026	-0.0648	2.9852	0.000146
chr5	9131308	9136066	*Dmtf1*	0.9683	0.8230	-0.1453	5.5848	0.00015
chr16	17845135	17856752	*Dgcr2*	0.9879	0.9334	-0.0545	5.4902	0.000182
chr16	94408716	94410833	*Ttc3*	0.9925	0.8888	-0.1037	14.8993	0.000244
chr4	59592318	59594396	*Hsdl2*	0.9801	0.9262	-0.0539	3.7039	0.000247
chr2	69741693	69746028	*Ppig*	0.9725	0.9024	-0.0701	3.5489	0.00025
chr7	1.42E+08	1.42E+08	*Chid1*	0.9876	0.9272	-0.0604	5.8899	0.000367
chr11	79242156	79244463	*Wsb1*	0.9588	0.8991	-0.0598	2.4516	0.000532
chr1	1.2E+08	1.2E+08	*Ptpn4*	0.9793	0.8764	-0.1028	5.9574	0.000597
chr10	83737933	83758134	*1500009L16Rik*	0.9938	0.8995	-0.0943	16.2830	0.000837
chr8	75051657	75052150	*Tom1*	0.9947	0.9301	-0.0646	13.2732	0.000883
chr11	58848702	58856061	*Gm12258*	1.0000	0.8671	-0.1329	> 150	0.000917
chr9	55858525	55859748	*Scaper*	1.0000	0.8226	-0.1774	> 150	0.001059
chr17	34905456	34905910	*Ehmt2*	0.9718	0.8790	-0.0928	4.2930	0.001613
chr14	56957103	56959715	*Zmym2*	0.9958	0.9412	-0.0546	14.0286	0.001684
chr5	1.1E+08	1.1E+08	*Fbrsl1*	0.9800	0.7715	-0.2085	11.4248	0.002646
chr5	3610288	3615075	*Pex1*	1.0000	0.8614	-0.1386	> 150	0.004794
chr9	54477528	54501351	*Dmxl2*	0.9831	0.9073	-0.0758	5.4917	0.005274
chr17	75540191	75544748	*Fam98a*	0.9778	0.8999	-0.0780	4.5140	0.0057
chr11	96773222	96776968	*Snx11*	0.9873	0.9256	-0.0617	5.8572	0.008094
**Mean:**				0.9838	0.8643	-0.1195	17.5918	
**Median:**				0.9877	0.8888	-0.0944	8.5959	

Appendix Table S4 – Splicing, CE and SE analysis on 50 permutations and the inversion of sample groups shows SEs are not due to random sampling.

Permutation Control Groups*	Differentially Spliced Exons	CEs	SEs
M323K_WT_1+M323K_WT_2+M323K_WT_3+M323K_WT_4	920	2	47
M323K_HOM_1+M323K_HOM_2+M323K_HOM_3+M323K_HOM_4	920	4	9
M323K_HOM_2+M323K_HOM_3+M323K_HOM_4+M323K_WT_3	38	0	0
M323K_HOM_1+M323K_WT_1+M323K_WT_2+M323K_WT_4	38	0	0
M323K_HOM_1+M323K_HOM_2+M323K_HOM_4+M323K_WT_4	25	0	1
M323K_HOM_3+M323K_WT_1+M323K_WT_2+M323K_WT_3	25	0	0
M323K_HOM_1+M323K_HOM_3+M323K_HOM_4+M323K_WT_4	25	0	2
M323K_HOM_2+M323K_WT_1+M323K_WT_2+M323K_WT_3	25	1	0
M323K_HOM_1+M323K_HOM_2+M323K_HOM_3+M323K_WT_1	24	0	0
M323K_HOM_4+M323K_WT_2+M323K_WT_3+M323K_WT_4	24	0	0
M323K_HOM_2+M323K_WT_1+M323K_WT_2+M323K_WT_4	24	1	0
M323K_HOM_1+M323K_HOM_3+M323K_HOM_4+M323K_WT_3	24	0	0
M323K_HOM_1+M323K_WT_1+M323K_WT_2+M323K_WT_3	19	0	0
M323K_HOM_2+M323K_HOM_3+M323K_HOM_4+M323K_WT_4	19	1	0
M323K_HOM_1+M323K_HOM_2+M323K_HOM_4+M323K_WT_2	17	0	0
M323K_HOM_1+M323K_HOM_2+M323K_HOM_3+M323K_WT_3	15	0	0
M323K_HOM_4+M323K_WT_1+M323K_WT_2+M323K_WT_4	15	0	0
M323K_HOM_2+M323K_HOM_3+M323K_HOM_4+M323K_WT_1	12	0	0
M323K_HOM_1+M323K_HOM_2+M323K_HOM_3+M323K_WT_4	7	0	0
M323K_HOM_1+M323K_HOM_3+M323K_HOM_4+M323K_WT_2	7	0	0
M323K_HOM_4+M323K_WT_1+M323K_WT_2+M323K_WT_3	7	0	0
M323K_HOM_3+M323K_WT_2+M323K_WT_3+M323K_WT_4	7	0	0
M323K_HOM_1+M323K_HOM_3+M323K_HOM_4+M323K_WT_1	5	0	0
M323K_HOM_2+M323K_WT_2+M323K_WT_3+M323K_WT_4	5	0	0
M323K_HOM_3+M323K_WT_1+M323K_WT_2+M323K_WT_4	2	0	0
M323K_HOM_1+M323K_HOM_2+M323K_HOM_3+M323K_WT_2	1	0	0
M323K_HOM_4+M323K_WT_1+M323K_WT_3+M323K_WT_4	1	0	0
M323K_HOM_3+M323K_HOM_4+M323K_WT_3+M323K_WT_4	51	0	0
M323K_HOM_1+M323K_HOM_4+M323K_WT_1+M323K_WT_4	11	0	0
M323K_HOM_2+M323K_HOM_3+M323K_WT_2+M323K_WT_3	11	0	0
M323K_HOM_1+M323K_HOM_2+M323K_WT_1+M323K_WT_3	11	1	0
M323K_HOM_3+M323K_HOM_4+M323K_WT_2+M323K_WT_4	11	0	0
M323K_HOM_2+M323K_HOM_4+M323K_WT_3+M323K_WT_4	11	0	0
M323K_HOM_2+M323K_HOM_3+M323K_WT_1+M323K_WT_3	9	0	0
M323K_HOM_1+M323K_HOM_4+M323K_WT_2+M323K_WT_4	9	0	0
M323K_HOM_1+M323K_HOM_2+M323K_WT_3+M323K_WT_4	9	0	0
M323K_HOM_3+M323K_HOM_4+M323K_WT_1+M323K_WT_2	9	0	0

M323K_HOM_2+M323K_HOM_3+M323K_WT_1+M323K_WT_4	9	0	0
M323K_HOM_2+M323K_HOM_3+M323K_WT_3+M323K_WT_4	7	0	0
M323K_HOM_1+M323K_HOM_4+M323K_WT_1+M323K_WT_2	7	0	0
M323K_HOM_3+M323K_HOM_4+M323K_WT_2+M323K_WT_3	6	0	0
M323K_HOM_1+M323K_HOM_2+M323K_WT_1+M323K_WT_4	6	0	0
M323K_HOM_2+M323K_HOM_3+M323K_WT_1+M323K_WT_2	4	0	0
M323K_HOM_1+M323K_HOM_4+M323K_WT_3+M323K_WT_4	4	0	0
M323K_HOM_1+M323K_HOM_2+M323K_WT_2+M323K_WT_4	4	0	1
M323K_HOM_2+M323K_HOM_3+M323K_WT_2+M323K_WT_4	3	0	0
M323K_HOM_3+M323K_HOM_4+M323K_WT_1+M323K_WT_4	21	0	1
M323K_HOM_2+M323K_HOM_4+M323K_WT_2+M323K_WT_4	18	0	0
M323K_HOM_1+M323K_HOM_3+M323K_WT_3+M323K_WT_4	16	0	0
M323K_HOM_2+M323K_HOM_4+M323K_WT_1+M323K_WT_2	16	0	0
M323K_HOM_1+M323K_HOM_3+M323K_WT_2+M323K_WT_4	14	0	1
M323K_HOM_2+M323K_HOM_4+M323K_WT_1+M323K_WT_4	2	0	0

** Samples included in the control group for each analysed permutation.*

Correct sample grouping (dark green); reverse sample grouping (light green); permutations with a distribution of 3:1 (yellow); permutations with a 2:2 assignment (red).

Appendix Table S5 – The relationship between constitutive, alternatively spliced and skiptic exons with TDP-43 binding.

	**Total**	**Overlap**	**Percent**
All exons in GENCODE vM12	744,786	37,276	5
All constitutive exons found in all samples	239,897	17,828	7.4
All cassette exons found in all samples	5,656	361	6.4
Significant cassette exons (FDR < 0.05)	260	49	18.8
Skiptic exons (control PSI > = 0.95; dPSI > = -0.05; FDR < 0.05)	47	31	66

Appendix Table S6 – Gene ontology results from differentially expressed genes in *RRM2mut* embryo brains, using GOseq.

Fibroblast line	***Mutation***	***Diagnosis***	***Age at onset***	***Site of onset****	***Gender***	***Age at biopsy***
TARDBP 1	G298S	ALS	62	LL	M	64
TARDBP 2	A382T	ALS	59	UL	F	62
TARDBP 3	A382T	ALS	25	LL	F	31
TARDBP 4	A382T	ALS	67	B	M	69
CTRL 1	-	Healthy	-	-	F	67
CTRL 2	-	Healthy	-	-	M	64
CTRL 3	-	Healthy	-	-	M	67
CTRL 4	-	Healthy	-	-	F	69

**B, bulbar; UL, upper limb; LL, lower limb*

Supplementary materials and methods

KEY RESOURCES TABLE

Reagent or Resource	Source	Identifier
**Antibodies**		
p62	Abcam	Cat# ab91526
TDP-43 – IHC	Novus Biologicals	Cat# 10782-2-AP
TDP-43 - iCLIP	Sigma	Cat#T1705
TDP-43 – WB	Proteintech	Cat#12892-1-AP
Ubiquitin	Santa Cruz	Cat#Sc-8017
**Chemicals**		
Trizol	Life Technologies	Cat#15596026
QIAzol	Qiagen	Cat#79306
1-Bromo-3-chloropropane	Sigma	Cat#B62404
Agarose	Thermo	Cat#16500500
TBE Buffer	National Diagnostics	Cat#EC-860
Ethidium bromide	Sigma	Cat#E1510
Dynabeads Protein A	Thermo	Cat#10002D
Igepal CA-630	Sigma	Cat#I8896
T4 PNK	NEB	Cat#M0201S
RNA ligase	NEB	Cat#M0204S
Pre-adenylated L3-App	Jernej Ule lab	NA
CircLigase	Epicentre	Cat#CL9021K
RNase I	Thermo	Cat#EN0601
Turbo DNase	Thermo	Cat#AM2238
Proteinase K	Sigma	Cat#3115887001
cOmplete Mini Protease inhibitor cocktail	Sigma	Cat#4693124001
ATP[γ-^{32}P]	Perkin Elmer	Cat#NEG502A250UC
BamHI	Thermo	Cat#FD0054
AMPure XP beads	Beckman Coulter	Cat#A63880
Triton X-100	Sigma	Cat#X100
TWEEN 20	Sigma	Cat#P9416
Prolong Anti-fade Mountant with DAPI	Thermo	Cat#P36962
Effectene	Qiagen	Cat#301425
**Commercial Assays**		
miRNeasy Mini Kit	Qiagen	Cat#217004
RNase-Free DNase Set	Qiagen	Cat#79254
RNA ScreenTape	Agilent	Cat#5067-5576
RNA ScreenTape Sample Buffer	Agilent	Cat#5067-5577
RNA ScreenTape Ladder	Agilent	Cat#5067-5578
D1000 ScreenTape	Agilent	Cat#5067-5582
D1000 ScreenTape reagents	Agilent	Cat#5067-5583
2X PCR Master Mix	Thermo	Cat#K0172
SuperScript III Reverse Transcriptase	Invitrogen	Cat#18080044
PowerUp SYBR Green Master Mix	Thermo	Cat#A25742
Accuprime Supermix 1	Thermo	Cat#12342010

DC Protein Assay	Bio-Rad	Cat#5000112
DAB-MAP kit	Ventana	Cat#760-124
NuPAGE 4-12% Bis-Tris gel	Invitrogen	Cat#NP0322
Novex 6% TBE-Urea gel	Invitrogen	Cat#EC6865
MEGAshortscript kit	Ambion	Cat#AM1354
Message Max T7 Arca Capped Message Transcript Kit	Cellscript	Cat#C-MMA60710
Polymerase Tailing Kit	Epicentre	Cat#PAP5104H
MEGAclear kit	Ambion	Cat#AM1908
DNA Extract All Reagents kit	Applied Biosystems	Cat#4403319
Expand Long Range dNTPack	Roche	Cat#4829034001
Zero-blunt PCR cloning kit	Invitrogen	Cat#K270020
**Software**		
Bash 4.1.2	https://www.gnu.org/software/bash/	https://www.gnu.org/software/bash/
R 3.3.2	https://www.r-project.org	https://www.r-project.org
Python 2.7.1	https://www.python.org	https://www.python.org/
GraphPad Prism	GraphPad Software	http://www.graphpad.com/scientificsoftware/prism/
SAMtools 1.3.1	(Li *et al*, 2009)	http://samtools.github.io/
Trim Galore! 0.4.1	https://www.bioinformatics.babraham.ac.uk/projects/trim_galore/	https://github.com/FelixKrueger/TrimGalore
STAR 2.4.2a	(Dobin *et al*, 2013)	https://github.com/alexdobin/STAR
Novosort 3.07.01	Novocraft	http://www.novocraft.com/
HTSeq	(Anders *et al*, 2015)	http://htseq.readthedocs.io/
DESeq2 1.14.1	(Love *et al*, 2014)	http://bioconductor.org/
DEXSeq 1.2	(Anders *et al*, 2012)	http://bioconductor.org/
SGSeq 1.8	(Goldstein *et al*, 2016)	http://bioconductor.org/
MEME v4.11.2	(Bailey *et al*, 2015)	
BedTools v2.24.0	(Quinlan & Hall, 2010)	http://bedtools.readthedocs.io/
PhyloP	(Pollard *et al*, 2010)	https://genome.ucsc.edu
bigWigSummary	https://genome.ucsc.edu	https://genome.ucsc.edu
PSI	(Katz *et al*, 2010)	N/A
tidyverse	http://www.tidyverse.org/	http://www.tidyverse.org/
Ensembl 82 mouse	(Cunningham *et al*, 2015)	http://sep2015.archive.ensembl.org/
GENCODE mouse 25	(Mudge & Harrow, 2015)	https://www.gencodegenes.org
iCOUNT	http://github.com/tomazc/iCount	icount.fri.uni-lj.si http://github.com/tomazc/iCount
**Published data**		
TDP-43 knockdown RNA-seq data	(Polymenidou *et al*, 2011)	https://www.ncbi.nlm.nih.gov/sra - PRJNA141971
Mouse embryonic brain TDP-43 iCLIP	(Rogelj *et al*, 2012)	icount.fri.uni-lj.si - 20100222_LUjt3 https://genome.ucsc.edu
Mouse embryonic brain TDP-43 iCLIP	(Rogelj *et al*, 2012)	icount.fri.uni-lj.si - 20091102_LUjt5 https://genome.ucsc.edu
**Deposited data**		
Raw data	This paper	SRA reference: SRP133158

RNA-seq datasets (SRA database, reference: SRP133158)

Tissue	Genotype	N	Read length	Avg uniquely mapped reads
MEFs	RRM2mut	3	50nt x 2	4-13M
	LCDmut	3	50nt x 2	10-13M
	TDP-43 shRNA	3	50nt x 2	7-12M
Embryonic head	RRM2mut	3	40nt x 2	26-48M
	LCDmut	3	40nt x 2	27-34M
	Double	3	40nt x 2	15-50M
Adult spinal cord	RRM2mut	4	75nt x 2	41-53M
	LCDmut	4	75nt x 2	45-58M
Embryonic Brain	RRM2mut	4	100nt x 2	31-36M

RNA-seq analyses

All RNA sequencing reads underwent adapter and quality trimming with Trim Galore! (0.4.1). Trimmed reads were then aligned to the mm10 mouse genome sequence using STAR (2.4.2a) with two-pass alignment. The resulting BAM files were sorted and duplicate reads were flagged using NovoSort (1.03.09).

Differential gene expression

Reads were assigned to genes in the Ensembl 82 release of the mm10 build (Cunningham *et al*, 2015) using HTSeq (Anders *et al*, 2015), ignoring duplicate reads. Differential gene expression was performed using DESeq2 (Love *et al*, 2014).

To assess the relationship between intron length and differential expression, the longest intron in each gene was found using annotations from GENCODE mouse release 25 (Mudge & Harrow, 2015) using a custom Python script (2.7.1). The unadjusted p-values from DESeq2 were converted into Z-scores and given the sign of the $\log_2$ fold change. Genes were ordered by signed Z-score and binned into groups of 200. The mean intron length and standard error of the mean was plotted for each group. RNA sequencing data from of TDP-43 knockdown in mouse adult striatum (Polymenidou *et al*, 2011) was processed using the same analysis pipeline and used as a positive control.

Differential splicing

Three different generations and qualities of sequencing data being were generated over the course of the study. Therefore the methods used to measure splicing changes have been tailored to each dataset. RNA sequencing allows for unambiguous assignment of splice events in the form of spliced junction reads. However, due to their rare occurrence compared to non junction-spanning reads, the number of junction reads detected in a sample and therefore the power to resolve differential splicing depends on the initial depth of sequencing, the length of sequencing reads and the expression level of the gene. Therefore for low depth sequencing data it is practical to instead infer splicing changes from quantifying read coverage across each exon and ignore junction information. This approach is exemplified by the DEXSeq package (Anders *et al*, 2012) which we used to estimate splicing changes in the embryonic fibroblast and head samples.

When sequencing depth and read length is increased it is possible to more accurately measure splicing variation with spliced junction reads alone. The cassette exon is a splicing variant comprised of three spliced junction reads: two flanking junctions that connect the flanking exons to the central cassette exon and a single parent junction that excludes it. By taking the ratio of the inclusion junction counts over the total number of junctions we can estimate the percent spliced in (PSI) of the cassette exon (Katz *et al*, 2010). By comparing samples across conditions we can estimate a ΔPSI - the difference in PSI between cases and controls. A positive ΔPSI indicates increased exon inclusion and negative ΔPSI indicates increased exon skipping.

Due the high depth and long read length of the *RRM2mut* embryonic brain and *LCDmut* adult spinal cord samples we used the SGSeq package (Goldstein *et al*, 2016). This creates a local splicing graph of connected spliced junction reads and determines the splicing events contained within. These events consist of cassette exons, retained introns, alternate 3' and 5' junctions, alternate first and last exons, and mutually exclusive exons and SGSeq allows for the possibility of multiple classes of splicing event to occur within the same interval. SGSeq then quantifies the number of reads in each sample that support each splice event and these counts can be used with DEXSeq.

Pie charts were created showing the proportions of different types of events in an analysis, breaking complex events down and counting the individual events separately. For the remaining splicing analyses the cassette exon splicing events were focused upon.

Annotation of splicing events

Due to the current interest in unannotated (novel or cryptic) splicing events, particularly those linked to TDP-43 depletion (Humphrey *et al*, 2017; Ling *et al*, 2015), there is a need for tools that identify and classify spliced junction reads that cannot be assigned to known transcripts. SGSeq has the option of incorporating novel junctions into its splicing graphs, giving equal weight to novel and annotated splicing events.

As transcript annotation progresses the number of novel splicing events will diminish over time, and for this reason we have chosen to define extreme splicing changes by the levels of inclusion rather than annotation, which will naturally include unannotated events. For cryptic exons we use percent spliced in (PSI) in our control samples and the delta PSI between mutants and controls to select cassette exon splicing events that are barely included in controls (PSI < 5%) and then more included in the mutants (dPSI > 5%). What we call "skiptic" exons are extreme cassette exon skipping events, where an exon that is apparently constitutive (PSI > 95%) is then skipped in the mutants (dPSI > 5%).

Motif analysis

Exons were flanked by 100nt either side and FASTA sequence extracted. Motif discovery was run using the MEME motif discovery tool (Bailey *et al*, 2015), restricting to 8 letter motifs. A set of exonic sequences not found to be differentially spliced were used as a background.

Functional analysis of extreme cassette splice events

Cassette exons and their parent introns were extracted from the SGSeq results. A per-nucleotide list of PhyloP conservation scores (Pollard *et al*, 2010) for the mouse aligned to 59 other vertebrates (mm10.60way.phyloP60way.bw) was downloaded from UCSC. Mean scores were calculated for each exon using bigWigSummary (UCSC). The extreme cassette exons were compared to all exons annotated in the GENCODE mouse release 25.

Cassette exon splicing can destabilise its host transcript with either its inclusion or exclusion leading to a downstream frameshift and the presence of premature termination codons (Lewis *et al*, 2003). To predict the functional consequences of exon inclusion or skipping on the host transcript a custom

script was written in R that predicted the upstream and downstream exons that flank the extreme cassette exons using both GENCODE annotation and the spliced reads in from the aligned RNA-seq samples. If both flanking exons were predicted to be in the coding sequence then the exon sequences were concatenated with and without the central exon and translated *in silico* in the predicted codon frame of the upstream exon. If skipping or inclusion of the central exon caused a frameshift and/or a premature stop codon this was noted.
To assess the correlation between the presence of an extreme cassette exon and changes in expression of its host gene, the proportion of genes that are significantly up- or downregulated at FDR < 10% was assessed in extreme cassette exons non-extreme cassette exons and as a control, genes with no cassette splicing expressed at a level at or greater than the most lowly expressed extreme exon gene. The proportions of up- and downregulated genes were compared between the control genes and the two groups of cassette exon containing genes with a binomial test in R.

iCLIP-seq analyses
Analysis of high-throughput iCLIP libraries was conducted using the iCount pipeline (http://icount.fri.uni-lj.si), mapping reads to mm10. Only uniquely-mapped sense reads from any dataset were used. All peak calling and false discovery rate correction was carried out as described in (Huppertz *et al*, 2014; König *et al*, 2010). Replicates with similar pentamer enrichment profiles and read counts were grouped for subsequent analysis. Pentamer counts and annotation of peaks to genes were provided by iCount. Pentamer analyses were conducted on 30nt intervals immediately surrounding the crosslinked site.

RNA maps were created for groups of cassette exons by quantifying per-nucleotide iCLIP coverage across the entire length of each parent intron that contains the splice sites of each cassette exon. To maximise potential coverage, all iCLIP replicates created were pooled together with TDP-43 iCLIP generated previously (Rogelj *et al*, 2012). Analysis was then restricted to 300nt around the parent intron splice sites and 300nt around the cassette exon splice sites. Per-nucleotide iCLUP coverage was defined as the number of overlaps with at least one iCLIP cluster at an individual nucleotdie divided by the total number of exon sequences. Due to variance in exon lengths, it was simply noted whether the exon overlapped with at least one iCLIP cluster and this is plotted as a proportion of all exons with a separate axis. The 20 cassette exons with the greatest total coverage are plotted individually.

To assess the dependence between iCLIP coverage and intron length, total TDP-43 iCLIP coverage across the entire length of genes was calculated and normalised to give a per-nucleotide coverage proportion. Genes were divided into those contained introns >100kb (see above) and to whether they were upregulated or downregulated in the *RRM2mut* compared to wildtype littermates. Coverage distributions were compared using a Mann-Whitney-Wilcoxon Test in R.

Statistical analyses
All differential expression results are significant at a Benjamini-Hochberg false discovery rate of 10%. All differential splicing results presented are significant at a false discovery rate of 1% unless specifically stated. Graphpad PRISM was used to perform ANOVA with Bonferroni correction and t-tests (two-sided) and significance in graphs is indicated as * ($p<0.05$), ** ($p<0.01$) and *** ($p<0.001$).
P values are here reported where unable to fit in figure legends:

Data and software availability
All sequencing data have been deposited in a public repository (Sequence Read Archive – SRA – www.ncbi.nlm.gov) upon publication.

All scripts to trim, align and perform differential expression and splicing analyses is available at https://github.com/plagnollab/RNASeq_pipeline . All code written to perform custom analyses and prepare figures for this manuscript is available at https://github.com/jackhump/Two_TDP-43_Mutant_Mice.

References

Anders S, Pyl PT & Huber W (2015) HTSeq--a Python framework to work with high-throughput sequencing data. *Bioinforma. Oxf. Engl.* **31:** 166–169

Anders S, Reyes A & Huber W (2012) Detecting differential usage of exons from RNA-seq data. *Genome Res.* **22:** 2008–2017

Bailey TL, Johnson J, Grant CE & Noble WS (2015) The MEME Suite. *Nucleic Acids Res.* **43:** W39-49

Cunningham F, Amode MR, Barrell D, Beal K, Billis K, Brent S, Carvalho-Silva D, Clapham P, Coates G, Fitzgerald S, Gil L, Girón CG, Gordon L, Hourlier T, Hunt SE, Janacek SH, Johnson N, Juettemann T, Kähäri AK, Keenan S, et al (2015) Ensembl 2015. *Nucleic Acids Res.* **43:** D662-669

Dobin A, Davis CA, Schlesinger F, Drenkow J, Zaleski C, Jha S, Batut P, Chaisson M & Gingeras TR (2013) STAR: ultrafast universal RNA-seq aligner. *Bioinforma. Oxf. Engl.* **29:** 15–21

Goldstein LD, Cao Y, Pau G, Lawrence M, Wu TD, Seshagiri S & Gentleman R (2016) Prediction and Quantification of Splice Events from RNA-Seq Data. *PloS One* **11:** e0156132

Humphrey J, Emmett W, Fratta P, Isaacs AM & Plagnol V (2017) Quantitative analysis of cryptic splicing associated with TDP-43 depletion. *BMC Med. Genomics* **10:** 38

Huppertz I, Attig J, D'Ambrogio A, Easton LE, Sibley CR, Sugimoto Y, Tajnik M, König J & Ule J (2014) iCLIP: protein-RNA interactions at nucleotide resolution. *Methods San Diego Calif* **65:** 274–287

Katz Y, Wang ET, Airoldi EM & Burge CB (2010) Analysis and design of RNA sequencing experiments for identifying isoform regulation. *Nat. Methods* **7:** 1009–1015

König J, Zarnack K, Rot G, Curk T, Kayikci M, Zupan B, Turner DJ, Luscombe NM & Ule J (2010) iCLIP reveals the function of hnRNP particles in splicing at individual nucleotide resolution. *Nat. Struct. Mol. Biol.* **17:** 909–915

Lewis BP, Green RE & Brenner SE (2003) Evidence for the widespread coupling of alternative splicing and nonsense-mediated mRNA decay in humans. *Proc. Natl. Acad. Sci.* **100:** 189–192

Li H, Handsaker B, Wysoker A, Fennell T, Ruan J, Homer N, Marth G, Abecasis G, Durbin R & 1000 Genome Project Data Processing Subgroup (2009) The Sequence Alignment/Map format and SAMtools. *Bioinforma. Oxf. Engl.* **25:** 2078–2079

Ling JP, Pletnikova O, Troncoso JC & Wong PC (2015) TDP-43 repression of nonconserved cryptic exons is compromised in ALS-FTD. *Science* **349:** 650–655

Love MI, Huber W & Anders S (2014) Moderated estimation of fold change and dispersion for RNA-seq data with DESeq2. *Genome Biol.* **15:** 550

Mudge JM & Harrow J (2015) Creating reference gene annotation for the mouse C57BL6/J genome assembly. *Mamm. Genome Off. J. Int. Mamm. Genome Soc.* **26:** 366–378

Pollard KS, Hubisz MJ, Rosenbloom KR & Siepel A (2010) Detection of nonneutral substitution rates on mammalian phylogenies. *Genome Res.* **20:** 110–121

Polymenidou M, Lagier-Tourenne C, Hutt KR, Huelga SC, Moran J, Liang TY, Ling S-C, Sun E, Wancewicz E, Mazur C, Kordasiewicz H, Sedaghat Y, Donohue JP, Shiue L, Bennett CF, Yeo GW & Cleveland DW (2011) Long pre-mRNA depletion and RNA missplicing contribute to neuronal vulnerability from loss of TDP-43. *Nat. Neurosci.* **14:** 459–468

Quinlan AR & Hall IM (2010) BEDTools: a flexible suite of utilities for comparing genomic features. *Bioinforma. Oxf. Engl.* **26:** 841–842

Rogelj B, Easton LE, Bogu GK, Stanton LW, Rot G, Curk T, Zupan B, Sugimoto Y, Modic M, Haberman N, Tollervey J, Fujii R, Takumi T, Shaw CE & Ule J (2012) Widespread binding of FUS along nascent RNA regulates alternative splicing in the brain. *Sci. Rep.* **2:** 603
