# Supplementary material for: Mice with endogenous TDP‐43 mutations exhibit gain of splicing function and characteristics of amyotrophic lateral sclerosis
Source: EMBO J. 2018 May 15;37(11):e98684. doi: 10.15252/embj.201798684 (PMC5983119; doi:10.15252/embj.201798684)
Supplement: Supplementary file 2 — Expanded View Figures PDF [file EMBJ-37-e98684-s002.pdf]

Expanded View Figures

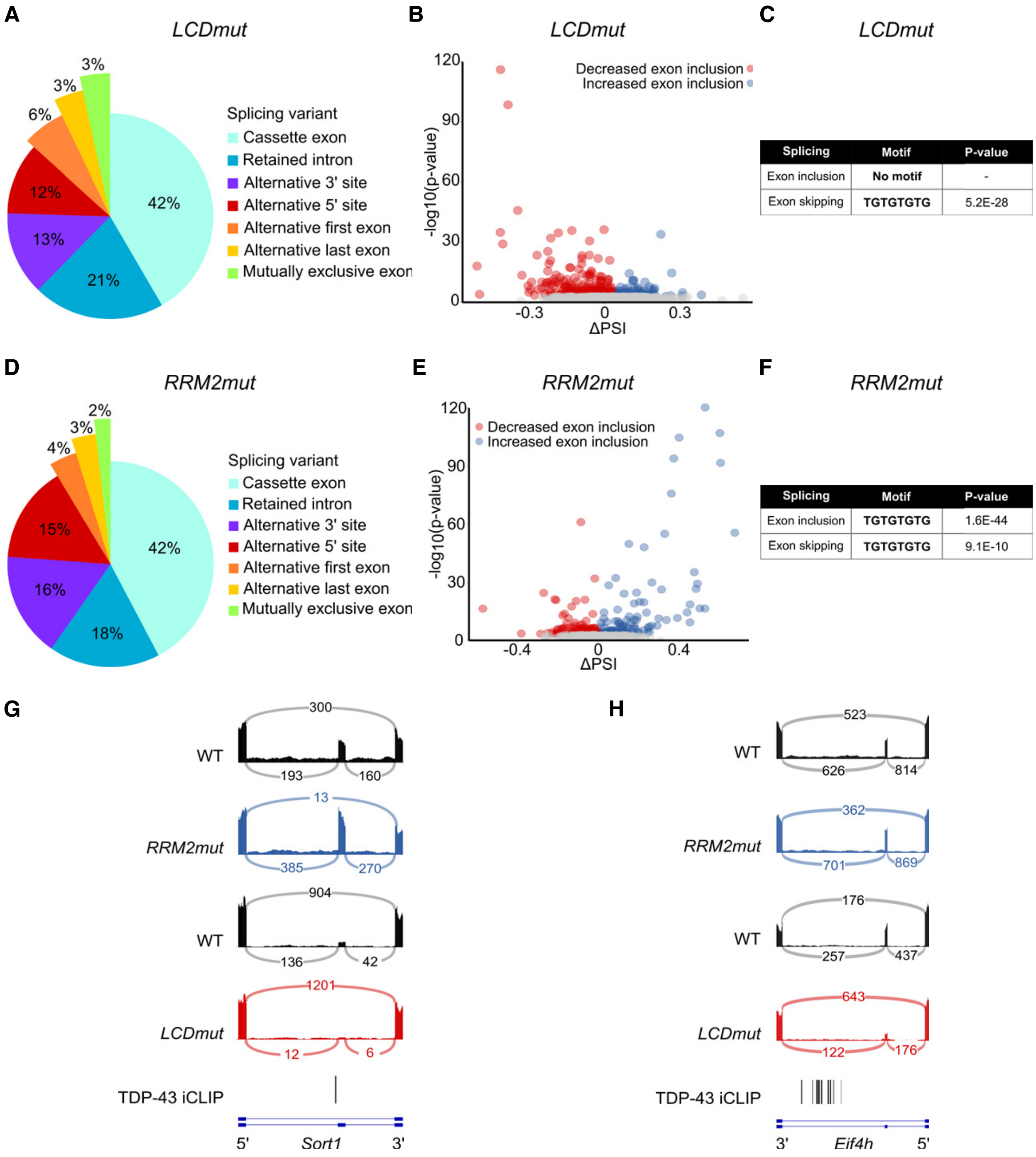

Figure EV1. Widespread splicing alterations in *LCDmut* and *RRM2mut*.

A–H Pie chart breakdown of differentially expressed splicing event classes in *LCDmut* (A) and *RRM2mut* (D). Volcano plots illustrate ΔPSI (*x*-axis) and *P*-value (*y*-axis) for cassette exons in *LCDmut* (B) and *RRM2mut* (E). Significant events (FDR < 0.01) are plotted in red (increased exon exclusion) and blue (increased exon inclusion). Non-significant events (FDR > 0.01) in grey. Motif enrichment in exons and flanking 100-bp intronic regions for significantly alternatively spliced cassette exons in *LCDmut* (C) and *RRM2mut* (F). Splicing changes in *Sort1* (G) and *Eif4h* (H) and their relation to TDP-43 binding sites are illustrated by sashimi plots and Tdp-43 iCLIP peaks.

© 2018 The Authors

© 2018 The Authors

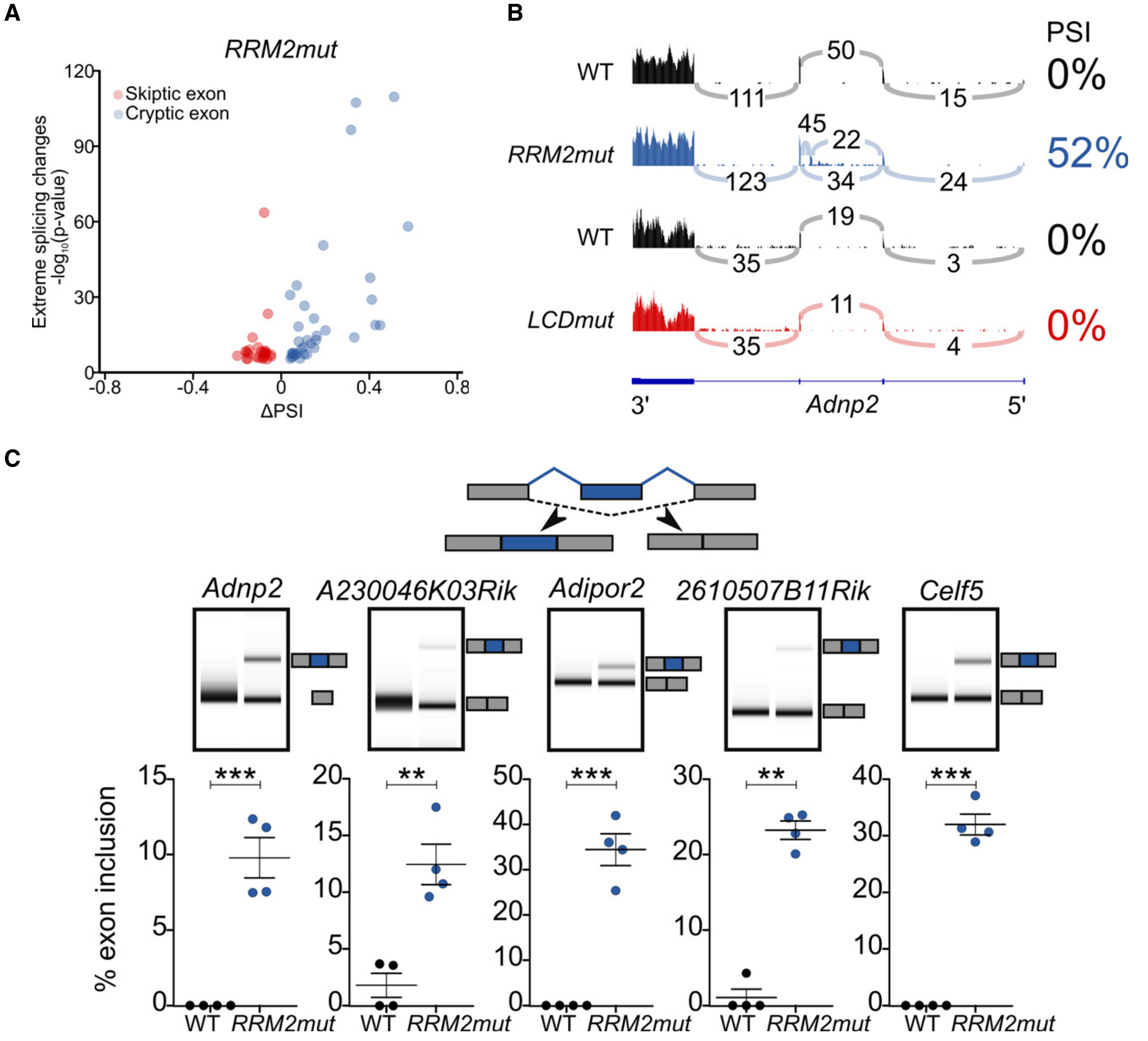

Figure EV2. Cryptic exons are widespread in *RRM2mut*.

A Volcano plot of *RRM2mut* RNA-seq shows significantly alternatively spliced exons that have either a PSI of < 0.05 and a ΔPSI of > 0.1 (CE, red) or a PSI of > 0.95 and a ΔPSI of < −0.1 (SE, blue). Twenty SEs occur in *RRM2mut*, and none are shared with *LCDmut* with one exception in *Tsn*, which is present also in compound heterozygous samples.

B Sashimi plots show presence of a CE in *Adnp2* in *RRM2mut*, absent in *LCDmut* and WT controls.

C Diagram illustrating CEs (top), representative lanes for acrylamide capillary traces (middle) and quantification (bottom) of RT–PCR (n = 4) validations for five CEs. *T*-test: *Adnp2* ****P* = 0.0008587; *A230Rik* ***P* = 0.003557; *Adipor2* ****P* = 9.439e-6; *2610Rik* ***P* = 0.005103; *Celf5* ****P* = 0.0004986. Plotted *P*-value: ***P* < 0.01; ****P* < 0.001. Mean and SEM plotted.

Source data are available online for this figure.

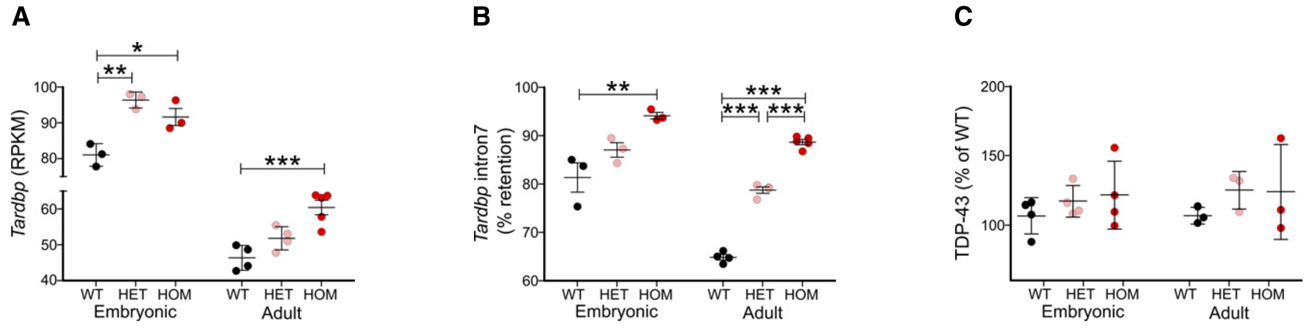

Figure EV3. *LCDmut* induces *Tardbp* upregulation.

A *Tardbp* mRNA levels from RNA-seq on WT and *LCDmut* embryo head and adult spinal cord show significant upregulation in *LCDmut*. Embryonic dataset ANOVA, $P = 0.0032$, $N = 3$; adult dataset ANOVA, $P = 0.0009$, $N = 4–5$; error bars: SD; Bonferroni multiple comparison tests are plotted as P-value: *$P < 0.05$; **$P < 0.01$; ***$P < 0.001$.

B % of *Tardbp* intron 7 retention is increased in *LCDmut* embryo head and adult spinal cord, in accordance with the autoregulation mechanism of *Tardbp* being shifted towards upregulating *Tardbp*. Embryonic dataset ANOVA, $P = 0.0113$, $N = 3$; adult dataset ANOVA, $P < 0.0001$, $N = 4–5$; error bars: SE; Bonferroni multiple comparison tests are plotted as P-value: **$P < 0.01$; ***$P < 0.001$.

C Quantification of TDP-43 protein levels in relation to β-actin in Western blots. Results are normalised to the mean of WT (100%). Embryonic dataset: ANOVA $P = 0.480$, $N = 4$; adult dataset: ANOVA $P = 0.491$, $N = 3$; error bars: SD.

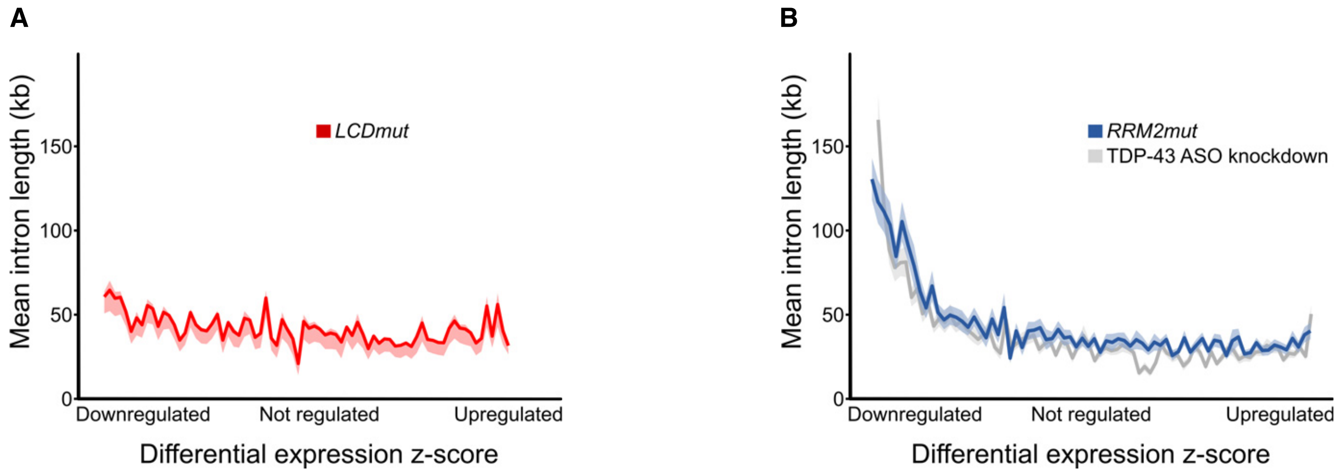

Figure EV4. *LCDmut* do not show TDP-43 molecular LOF features.

A, B In RNA-seq data from *LCDmut*, genes were ranked for gene expression based on signed z-score (x-axis), and the mean intron length for groups of 200 genes was plotted (y-axis), showing no enrichment of long intron genes in downregulated transcripts (A), unlike in *RRM2mut* where data show an enrichment of long intron genes in downregulated transcripts from *RRM2mut* (blue) and previously published data from adult striatum TDP-43 knockdown (grey; Polymenidou *et al*, 2011) (B). Ribbon plots show mean ± standard error of the mean (shaded regions).

 © 2018 The Authors
