# Supplementary figures and images for: Mice with endogenous TDP‐43 mutations exhibit gain of splicing function and characteristics of amyotrophic lateral sclerosis
Source: EMBO J. 2018 May 15;37(11):e98684. doi: 10.15252/embj.201798684 (PMC5983119; doi:10.15252/embj.201798684)

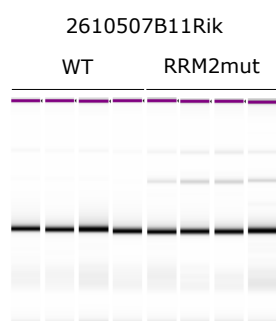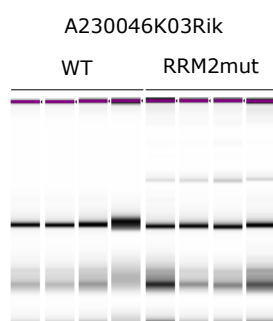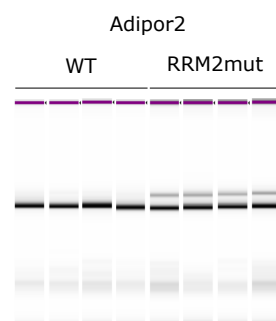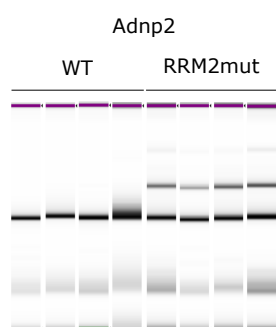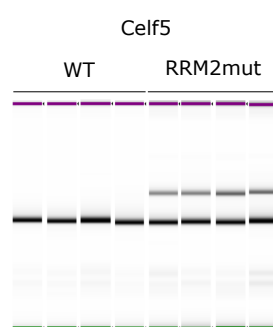

Supplement: Supplementary file 3 — Source Data for Expanded View [file EMBJ-37-e98684-s006.zip › Source_Data_EV_2.pdf]

***RRM2mut CFTR***

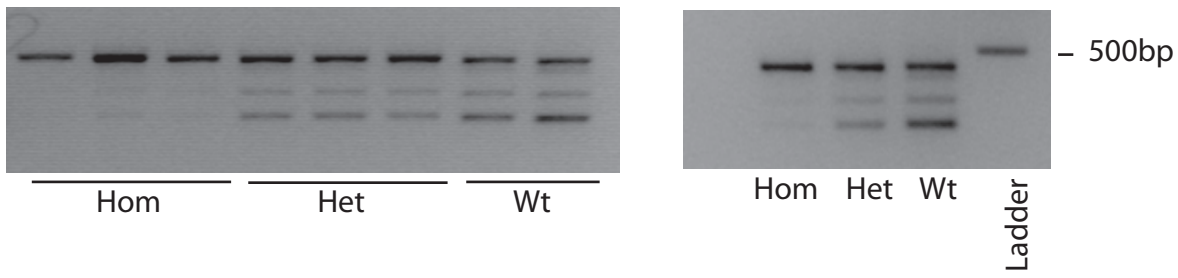

***LCDmut CFTR***

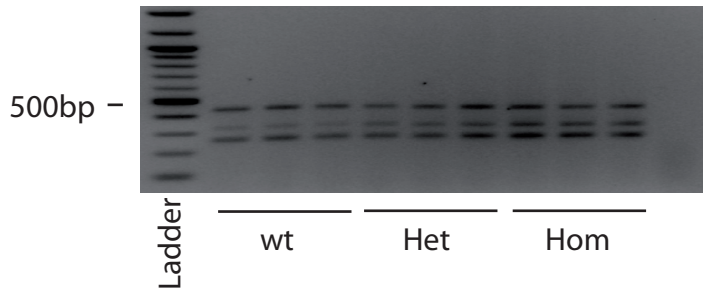

***Sort1 RRM2mut***

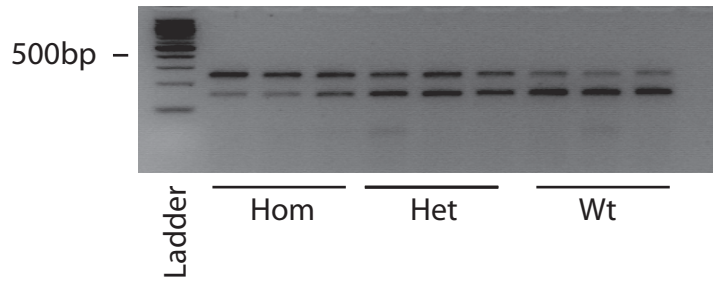

***Sort1 LCDmut***

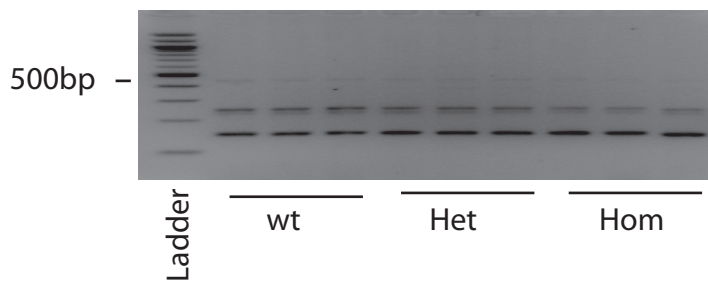

Supplement: Supplementary file 5 — Source Data for Figure 1 [file EMBJ-37-e98684-s003.pdf]

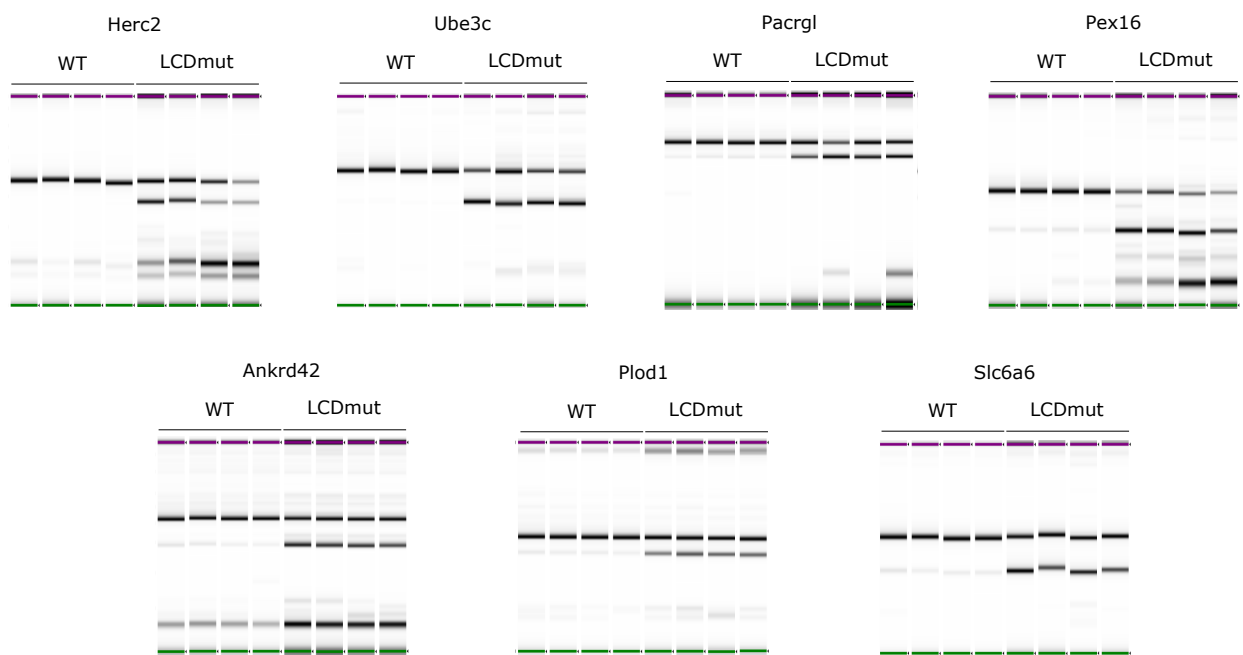

Supplement: Supplementary file 6 — Source Data for Figure 3 [file EMBJ-37-e98684-s004.pdf]

***Sort1***

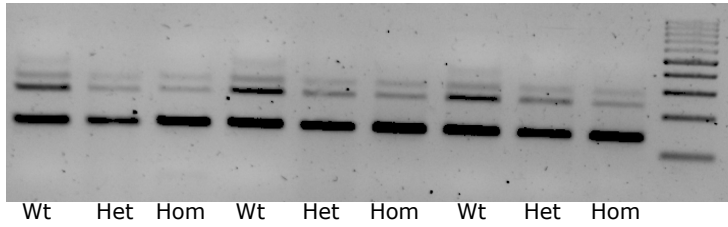

***Pacrgl***

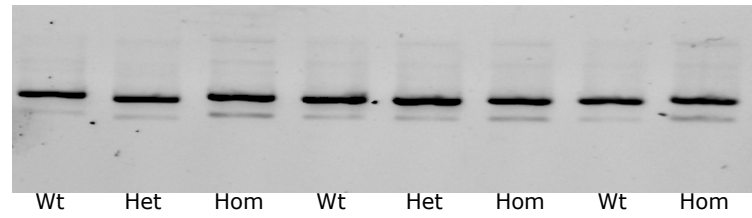

***Pex16***

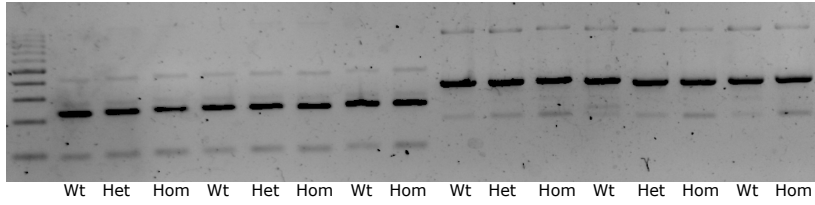

***Ube3c***

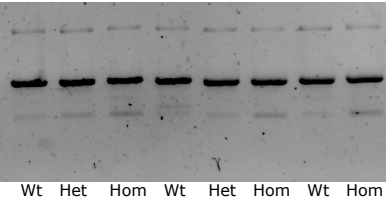

**CFTR assay on MEFs**

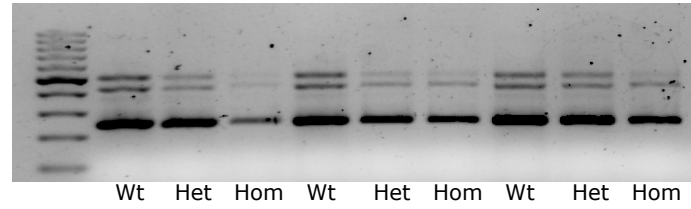

***Eif4h***

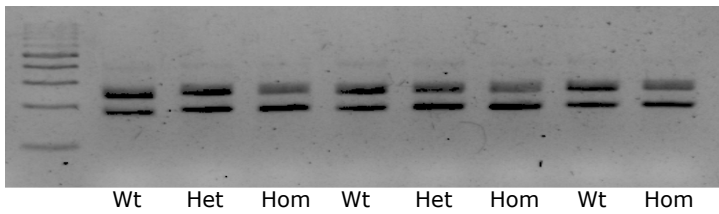

***Kcnip2***

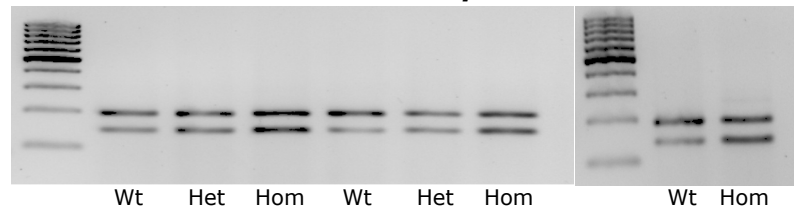

***SLC6A6***

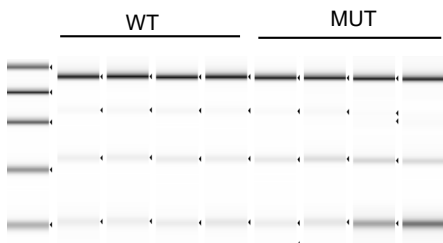

***PLOD1***

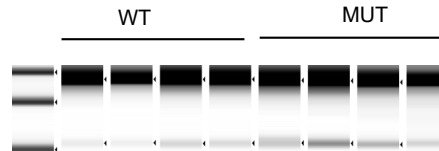

Ladder: 100bp ladder

Supplement: Supplementary file 7 — Source Data for Figure 7 [file EMBJ-37-e98684-s005.pdf]
